# YOLIP: An Enhanced Framework for UAV-Assisted Wildlife Monitoring Based on YOLO Integrated with the CLIP Model

**DOI:** 10.3390/s26113436

**Published:** 2026-05-29

**Authors:** Ruiheng Hu, Yiwei Chen, Kejia Xu, Leyan Zhang, Chengyang Yue, Hao Pi, Xuhua Chen, Xiaoyong Lin

**Affiliations:** 1 Portland College, Nanjing University of Posts and Telecommunications, Nanjing 210023, China; p23000323@njupt.edu.cn (R.H.); p24000206@njupt.edu.cn (Y.C.); p23000318@njupt.edu.cn (L.Z.); p23000321@njupt.edu.cn (C.Y.); p23000410@njupt.edu.cn (H.P.); p24000423@njupt.edu.cn (X.C.); 2 School of Communications and Information Engineering, Nanjing University of Posts and Telecommunications, Nanjing 210003, China; b22011305@njupt.edu.cn

**Keywords:** wildlife monitoring, object detection, feature alignment, multi-scale representation, cross-modal learning, real-time inference

## Abstract

UAV-based wildlife monitoring encounters tremendous challenges posed by complex environments, such as the extremely low proportion of effective targets in aerial images and variations in remote sensing scales. This paper presents a novel fusion framework named YOLIP, which integrates a detection head with semantic perception capabilities and an implicit feature adjustment module to boost detection accuracy and feature representation ability. Specifically, this paper redesigns the detection head to enable it to simultaneously learn spatial positioning and semantic features, thereby achieving more reliable extraction of regional features. The implicit feature modulation module introduces a dual-path fusion mechanism, which elevates the feature quality through geometric–semantic fusion, thereby improving the consistency and robustness of the detection. Furthermore, this paper also develops an asynchronous scheduling strategy, which can selectively execute computationally intensive operations to achieve computational optimization, enabling this framework to adapt to actual detection scenarios based on unmanned aerial vehicles. In this study, we conducted numerous experiments on the self-built drone wildlife dataset as well as the publicly available aerial wildlife dataset. Theresults demonstrate that compared with existing detection models, YOLIP improves mAP@0.5 by 11.6% while maintaining an efficient inference speed, achieving an improvement in detection performance. In addition, cross-dataset evaluation verifies the stable performance and generalization capability of the proposed method across multiple real-world scenarios.

## 1. Introduction

Effective monitoring of endangered wild animals in complex natural environments is a major challenge faced in the field of ecological protection. Due to its sparse distribution, wide coverage area, and significant differences in habitats, traditional monitoring methods relying on manual patrols or fixed camera systems have limitations in terms of real-time monitoring, including limited coverage range and insufficient time continuity [1]. With the rapid development of drone technology, large-scale dynamic monitoring based on continuous video streams has gradually become possible, providing new opportunities for real-time ecological observation [2].

However, accurately detecting and identifying wildlife targets from the perspective of drones remains a significant challenge. Animal targets captured from an aerial viewpoint typically occupy only a small number of pixels in the image, making it difficult for models to extract key features from them [3]. Complex environmental factors such as changes in lighting, occlusion, and background noise can also significantly degrade detection performance. Most existing methods still rely on closed-set training, limiting the model’s ability to generalize to unseen species in real-world scenarios. To address these challenges, researchers have adapted the YOLO series of models for UAV target detection, achieving a good balance between detection speed and accuracy [4,5,6]. However, in UAV-assisted wildlife monitoring scenarios, due to the aforementioned challenges, the performance of such methods remains limited in terms of small-target detection and adaptation to dynamic environments [7,8,9]. Especially when the ground sampling distance (GSD) increases with increase in flight altitude, the pixels occupied by each animal target will decrease, making it difficult to distinguish the texture, outline, and specific details of the category. In this situation, the degradation of early-stage features may lead to detection errors, and these errors cannot be compensated for by the subsequent recognition module.

In recent years, vision-language models represented by CLIP have demonstrated strong zero-shot and open-vocabulary recognition capabilities by aligning visual and textual representations in a shared embedding space. These characteristics make CLIP well suited for recognition tasks involving unseen categories [10,11,12,13]. However, CLIP itself lacks precise object localization capabilities and often incurs high computational costs when directly applied to dense visual scenes [14,15,16,17,18].

To overcome these limitations, recent studies have explored the approach of combining YOLO with CLIP. That is, by leveraging the precise localization capability of YOLO and the semantic generalization ability of CLIP, the performance of the object detection task can be improved. Among these studies, YOLO-World stands out as a representative work in this field. This method introduces a Visual-Language Path Aggregation Network (RepVL-PAN) and a region-text contrastive learning mechanism, achieving fundamental alignment of cross-modal features. The resulting model combines both precise localization and generalization capabilities [19]. Driven by this research paradigm, subsequent methods have further explored cross-modal representation enhancement strategies. YOLOE extends prompt-based detection capabilities while maintaining the existing inference overhead [20]. Uni-YOLO enhances the model’s robustness in cluttered backgrounds by establishing a CLIP-guided feature alignment mechanism [21]. CLIP-YOLO, on the other hand, replaces the traditional classification head with semantic embeddings and combines attention mechanisms to elevate the expressive power of visual features [22]. Mamba-YOLO-World further proposed a fusion mechanism based on the State Space Model (SSM). This mechanism combines linear computational complexity with a global receptive field, effectively enhancing feature interactions and improving the model’s generalization performance [23]. Furthermore, research has shown that embedding CLIP into the information of YOLOv8 during its training process can enhance the model’s data efficiency and stability. Especially in cases where the training data are limited, this approach demonstrates significant advantages [24].

Despite these advancements, existing drone wildlife detection methods and the YOLO-CLIP fusion approach still have the following limitations. Existing YOLO-CLIP fusion methods mainly focus on general visual–semantic alignment or open-category recognition. However, the geometric mismatch problem between the proposals generated by the detector and the CLIP-style visual input has not been fully resolved. Furthermore most existing YOLO–CLIP fusion methods are designed for image-level object detection or general open-category recognition tasks. In contrast, drone-based wildlife monitoring typically relies on continuous video streams, where changes in object appearance between adjacent frames are relatively slow. In this scenario, performing computationally intensive semantic recognition operations on every frame would inevitably result in redundant computations and reduce the model’s practical deployment efficiency on resource-constrained drone platforms. Therefore, a perception framework for UAV applications should not only focus on improving detection and recognition accuracy but also incorporate frame-level inference efficiency into the model design.

Furthermore, wildlife monitoring places higher demands on the synergy between high-recall target localization and semantic refinement. In aerial images, small-scale targets, partial occlusions, and complex background interference can easily lead to missed detections; once candidate regions are not generated correctly, it is typically difficult to compensate for this error in the subsequent semantic recognition stage. Therefore, reliable candidate region generation should be prioritized before semantic classification; simultaneously, the generated candidate regions need to be further refined in both geometric and semantic spaces to reinforce the stability of the final recognition.

To address these limitations, this paper proposes a hierarchical perception framework, YOLIP. This framework involves a YOLO foreground extraction branch with high recall rate and integrates YOLOv11 and CLIP through a unified cross-modal alignment process. The proposed YOLIP head converts the detector features into language-aligned proposal embeddings, thereby strengthening the connection between the localization based on YOLO and the semantic representation based on CLIP. The Interactive Fusion Module (IFM) further refines the candidate regions through geometric normalization and semantic alignment, reducing the distortion of the proposals and the mismatch of cross-modal representations. Furthermore, a frame-level asynchronous scheduling strategy was introduced, which decoupled the high-frequency positioning from the low-frequency semantic recognition, thereby reducing redundant computations in the continuous drone video stream.

The key contributions of this research are presented as follows:

- We propose a structured YOLOv11–CLIP model, which divides the process of object detection and semantic understanding. This framework has the ability to run in real-time and handle open vocabulary tasks.
- We design a YOLIP head with semantic perception capabilities, which is used to generate language-related proposal embeddings from the features of the detector. This enables a more consistent interface between the localization based on YOLO and the recognition based on CLIP.
- We introduce an Interactive Fusion Module (IFM), which optimizes the candidate regions through geometric standardization and semantic alignment. This helps to reduce the distortion of the proposal and the mismatch of cross-modal representations.
- We develop an asynchronous scheduling method aimed at reducing redundant semantic inference in continuous drone video streams, which increases throughput and enhances the performance of the existing video streaming system.

## 2. Hierarchical Perception Framework

This study proposes a hierarchical perception framework that is suitable for wildlife monitoring in complex natural environments. By integrating detection, recognition, and decision-making into a unified workflow, the framework enables accurate semantic mapping of visual observations used for category-level forecasts.

**Figure 1:**
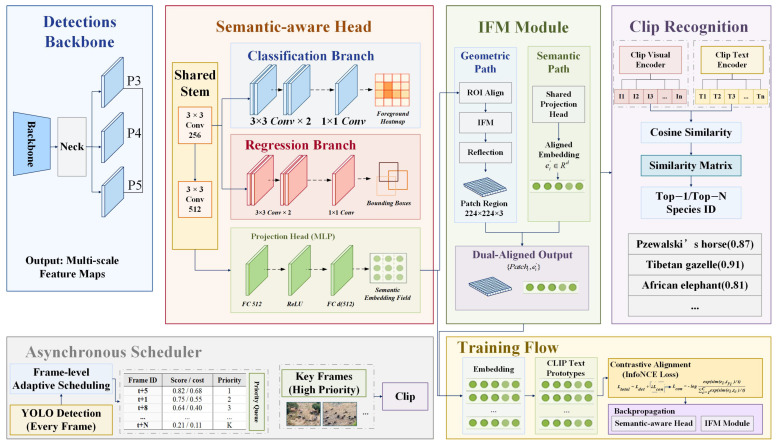
Hierarchical perception framework diagram.

Specifically, this system combines the real-time detection capability of YOLOv11 with the open vocabulary recognition capability of the CLIP model. By combining these complementary strengths, the framework achieves robust performance under small-sample and zero-shot scenarios. As shown in Figure 1, the proposed system consists of three core elements:

(1) A real-time object detection module based on YOLOv11, responsible for efficiently locating candidate regions in complex backgrounds;
(2) A semantic recognition module based on CLIP, which performs zero-shot classification via the alignment of visual features and text embeddings;
(3) A multi-modal fusion and decision module, which integrates detection and semantic data is utilized to generate ultimate forecasts.

To bridge the representational gap between YOLOv11 and CLIP, we introduce a semantic-aware detection head, termed the YOLIP Head, along with an Interaction Fusion Module (IFM). The YOLIP Head acts as a semantic projection interface, transforming detection features into language-aligned proposal embedding and establishing an initial alignment of visual features with the shared semantic domain. On top of this, the IFM performs further cross-modal alignment through a contrastive learning objective, refining the coherence between visual and textual depictions.

Together, this hierarchical alignment mechanism enables robust cross-modal representation and notably boosts generalization in situations with limited data and zero-shot cases.

Furthermore, the framework incorporates prompt engineering and feature normalization approaches to promote semantic coherence and steady the training procedure. Overall, the proposed framework establishes an efficient and scalable paradigm for multi-modal perception within real-world situations.

### 2.1. YOLOv11-Based Object Detection

In the first stage, this paper employs the lightweight YOLOv11n as the base detector to efficiently locate candidate regions from the drone images. Compared with earlier versions of YOLO, YOLOv11 introduces a more powerful design for feature extraction and spatial feature aggregation.

**Figure 2:**
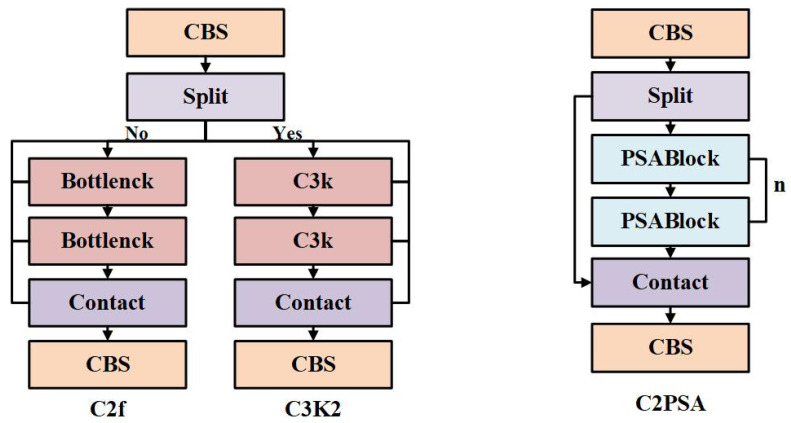
Schematic diagram of key architectural improvements in YOLOv11: C3k2 and the C2PSA attention module.

This is particularly important for UAV wildlife monitoring. The C3K2 module enhances the local feature extraction capability with lower computational overhead [6]. It introduces C3k blocks to better preserve local spatial details and multi-scale features. Its structure is shown in Figure 2.

These characteristics are highly relevant to UAV wildlife monitoring tasks, as the ground sample distance affected by the flight altitude means that animal targets are typically represented as small-scale targets in the images. Therefore, in this paper, YOLOv11n is selected as the location branch of YOLIP to achieve a good balance between detection accuracy and computational efficiency. At the same time, a relatively low confidence threshold is adopted to maintain a high recall rate, ensuring that sufficient foreground animal candidate regions are reserved for subsequent semantic recognition based on CLIP and IFM feature refinement.

### 2.2. CLIP Semantic Classification

In the recognition stage, the multi-modal pre-trained model CLIP is employed to perform semantic classification of candidate regions. CLIP maps visual and textual inputs into a shared embedding space through contrastive learning, enabling open-vocabulary recognition.

Specifically, each candidate region is encoded by the CLIP visual encoder, while category descriptions are converted into text embedding using prompt templates. The semantic similarity between image and text embedding is computed via cosine similarity, followed by SoftMax normalization to obtain classification probabilities.

This mechanism allows the system to generalize to unseen categories without additional training, making it particularly suitable for wildlife monitoring scenarios.

### 2.3. Overview: A Semantic-Aware Proposal Framework via Contrastive Alignment

YOLIP is a semantic-aware detection framework that transforms dense visual features into language-aligned proposal embeddings via contrastive learning. The study does not merely connect a detector to a classifier; it instead reformulates detection as semantic proposal generation. The overall process can be formulated as follows.

Let $I\in R^{H\times W\times 3}$ denote an input frame. A YOLOv11 backbone extracts multi-scale feature maps $F^{\left(s\right)}\in R^{H_{s}\times W_{s}\times C}$ at strides s∈8,16,32, where the number of channels is compressed to C=256.

These features are fed into a novel YOLIP Head, which projects every spatial cell into a CLIP-aligned semantic embedding field $E^{\left(s\right)}$ via a shared function $g_{\phi }$.

From this field, we extract a set of semantic-aware proposals $P={\left(B_{i},e_{i}\right)}_{i=1}^{N_{p}}$, where each bounding box $B_{i}$ is paired with a pre-aligned embedding $e_{i}\in R^{d}$. A dual-pathway Intermediate Feature Mapping (IFM) module then refines these proposals. Its geometric pathway normalizes each region to a fixed canonical size, while its semantic pathway produces a refined embedding $e_{i}^{\prime }$.

Finally, $e_{i}^{\prime }$ is matched against frozen CLIP text prototypes $t_{c}$ via cosine similarity. The entire framework is driven by a unified InfoNCE contrastive objective:

(1) $$L_{con}=-\frac{1}{N}\sum_{i=1}^{N}\log\frac{\exp(\mathrm{sim}\left(e_{i}^{\prime },t_{y_{i}}\right)/\tau )}{\sum_{c=1}^{C}\exp\left(\mathrm{sim}\left(e_{i}^{\prime },t_{c}\right)/\tau \right)},$$

where *N* is the number of valid proposals in a mini-batch, *C* denotes the number of text prototypes, τ is the temperature coefficient.

In order to jointly optimize the foreground positioning and semantic alignment, the overall training objective of YOLIP is set as:

(2) $$L_{total}=\lambda_{box}L_{box}+\lambda_{dfl}L_{dfl}+\lambda_{cls}L_{cls}+\lambda_{con}L_{con}$$

Here, $L_{box}$ represents the bounding box regression loss, $L_{dfl}$ is the distribution focus loss used for bounding box refinement, and $L_{cls}$ is responsible for supervising the classification of foreground proposals. These losses related to detection will optimize the localization branch of YOLOv11, thereby generating accurate and highly recallable animal proposals. In contrast, $L_{con}$ represents the constructed proposals in association with the semantic prototypes based on CLIP. These weight coefficients $\lambda_{box}$, $\lambda_{dfl}$, $\lambda_{cls}$, and $\lambda_{con}$ respectively determine the relative importance of localization learning, bounding box refinement, foreground proposal classification, and semantic alignment.

Overall, YOLIP can be viewed as a semantic proposal generation framework: detection provides spatial grounding, while contrastive alignment embeds language-level semantics directly into each proposal.

#### 2.3.1. YOLIP Head (YH): Generating Language-Aligned Proposal Embedding

**Figure 3:**
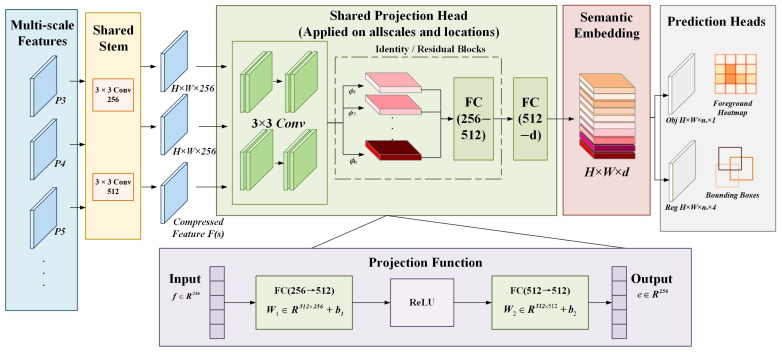
Generating language-aligned proposal embedding structure.

The YOLIP head extends the traditional YOLO detection head by introducing a semantic projection branch. Unlike standard YOLO heads that terminate in closed-set class logits, our design outputs dense semantic embeddings that are natively comparable with CLIP text prototypes. The structure diagram is shown in Figure 3.

Formally, let $F^{\left(s\right)}\in R^{H_{s}\times W_{s}\times C}$ denote the feature map at scale s(P3,P4,P5), compressed to C=256 via a 3×3 convolution. A shared projection function $g_{\phi }:R^{C}\to R^{d}$ is applied densely across all scales and spatial positions:

(3) $$E^{\left(s\right)}=g_{\phi }\left(F^{\left(s\right)}\right)\in R^{H_{s}\times W_{s}\times d},$$

where $g_{\phi }$ is a two-layer MLP:

(4) $$g_{\phi }\left(f\right)=W_{2}\cdot \sigma \left(W_{1}\cdot f+b_{1}\right)+b_{2},$$

with $W_{1}\in R^{512\times 256},W_{2}\in R^{512\times 512}$, ReLU activation σ, and output dimension d=512 that match CLIP ViT-B/32. The semantic embedding field is denoted as $E^{\left(s\right)}$.

On this field, two lightweight predictors operate. A foreground heatmap head produces a class-agnostic object score via a 1×1 convolution followed by a sigmoid:

(5) $$H^{\left(s\right)}=\sigma \left({\mathrm{Conv}}_{1\times 1}\left(E^{\left(s\right)}\right)\right)\in {\left[0,1\right]}^{H_{s}\times W_{s}\times 1}$$

A parallel bounding box regression head (anchor-free) predicts the coordinates.

For each high-confidence foreground location, we extract a proposal $B_{i}=\left(x_{i},y_{i},w_{i},h_{i}\right)$ and pool its embedding from $E^{\left(s\right)}$ using RoIAlign:

(6) $$e_{i}=\mathrm{RoIAlign}\left(E_{s},B_{i}\right)\in R^{d}$$

The output is a set of semantic-aware proposals:

(7) $$P={\left\{\left(B_{i},e_{i}\right)\right\}}_{i=1}^{N_{p}}$$

Crucially, the projection parameters ϕ are shared across all scales (*P*3–*P*5) and all spatial positions. This weight-sharing enforces scale-invariant semantics and reduces overfitting—A critical advantage in data-limited regimes.

In essence, the YOLIP Head transforms the detection backbone’s visual features into a language-ready proposal set, where each proposal already carries a CLIP-aligned semantic embedding.

#### 2.3.2. IFM: Interaction Fusion Module

The intermediate directly feeding YOLO proposals into CLIP leads to a dual domain gap: geometric (arbitrary region sizes vs. fixed 224×224 input) and semantic (detection-optimized local features vs. globally aligned CLIP embedding).

**Figure 4:**
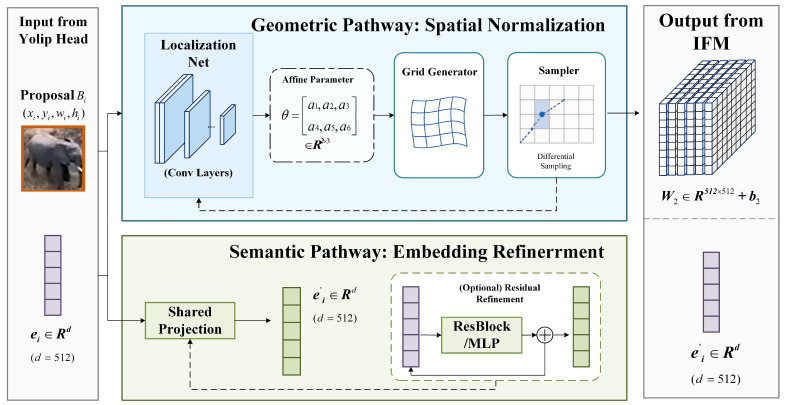
Interaction Fusion Module (IFM) architecture.

The Interaction Fusion Module (IFM) module effectively fills these two gaps through a dual-path architecture. The overall architecture of the system is shown in Figure 4.

Geometric Normalization Pathway. Given each candidate region $B_{i}$, a learnable spatial transformer estimates an affine transformation parameter $\Theta_{i}$. A grid generator and a differentiable sampler with reflection padding then warp the region to a canonical resolution:

(8) $$P_{i}=\mathrm{Warp}\left(B_{i},\Theta_{i}\right)\in R^{224\times 224\times 3}$$

Unlike naive resizing, this learned transformation adapts to object geometry and reduces spatial distortion.

Semantic Refinement Pathway. In parallel, the pre-aligned embedding $e_{i}$ is further refined through a weight-shared projection head $g_{\phi }^{\prime }$, which shares parameters with $g_{\phi }$ in the YOLIP Head:

(9) $$e_{i}^{\prime }=g_{\phi }^{\prime }\left(e_{i}\right)\in R^{d}$$

Weight-tying ensures that $e_{i}^{\prime }$ inhabits the exact same representational space as $e_{i}$, preventing feature drift and stabilizing cross-module training. The outputs of both pathways are jointly forwarded. The normalized patch $P_{i}$ enters CLIP’s visual encoder $f_{\mathrm{vis}}$, while $e_{i}^{\prime }$ is compared against frozen text prototypes $t_{c}$:

(10) $$s_{i,c}=\cos\left(e_{i}^{\prime },t_{c}\right)=\frac{e_{i}^{\prime }\cdot t_{c}}{∥e_{i}^{\prime }∥\;∥t_{c}∥}$$

During training, $e_{i}^{\prime }$ is supervised by the same InfoNCE loss $L_{\mathrm{align}}$ applied to $e_{i}$, creating a cascaded alignment mechanism: YOLIP Head provides coarse semantic initialization; IFM achieves precise optimization. Geometric standardization ensures spatial compatibility, while semantic optimization guarantees the accuracy of representation.

Combining these two paths can ensure that the visual markers entering CLIP not only have the correct spatial form, but also occupy the appropriate area in the semantic representation space.

## 3. Execution Framework and Throughput Analysis

### 3.1. Resource-Constrained Dual-Model Execution Mode

In the serial mode, each input frame will successively go through four steps: YOLOv11 detection, region proposal extraction, CLIP encoding, and result fusion. The total latency per frame is therefore the sum of these stages. Accordingly, the end-to-end frame rate (FPS) can be defined as follows: To maximize the efficiency of the YOLIP framework under the constraint of limited computing resources, two execution modes were designed: the serial mode and the asynchronous batch processing mode. As shown in Table 1, the asynchronous mode increases the throughput by overlapping computations between different stages.

(11) $${FPS}_{e2e}=\frac{1}{T_{yolo}+T_{clip}+nT_{post}}$$

where $T_{yolo}$ denotes the inference time of YOLOv11, $T_{clip}$ is the total CLIP encoding time over all proposals, $T_{post}$ represents the average post-processing time per proposal, and n is the number of proposals generated by the YOLIP Head.

To enhance the reasoning efficiency, we further proposed an asynchronous batch execution architecture. In this design, the detection model (YOLOv11) and the semantic encoder (CLIP) execute independently in separate CUDA streams, thereby achieving parallel computing and reducing the idle time caused by the high latency of CLIP.

Meanwhile, proposals from consecutive frames are dynamically aggregated into batches of size B before being processed by CLIP. This batching strategy significantly strengthens GPU utilization.

Under this mechanism, the average CLIP processing time per frame is reduced to $T_{clip}^{\left(B\right)}/B$, where $T_{clip}^{\left(B\right)}$ denotes the total processing time for a batch. The overall system throughput is determined by the slowest stage in the pipeline:

(12) $$Throughput=\frac{1}{\max\left(T_{\mathrm{yolo}},\frac{T_{\mathrm{clip}}^{\left(B\right)}}{B}\right)}$$

This asynchronous design advances hardware utilization by overlapping computation across stages and is particularly suitable for latency-insensitive scenarios such as offline video analysis.

### 3.2. Frame-Level Scheduling and Asynchronous Inference Optimization for Video Streaming

For the continuous video streams generated by drone patrols, we designed a frame-level adaptive scheduling strategy. We separate the high-frequency lightweight target localization from the low-frequency candidate region semantic recognition. The lightweight detection branch runs at the original video frame rate and is used for real-time monitoring and short-term tracking. The semantic recognition branch based on CLIP is activated in an event-triggered manner. Specifically, semantic recognition is triggered only when a target appears, disappears, or changes beyond a predefined threshold.

To quantitatively evaluate the performance improvement brought by the scheduling strategy, we defined a scheduling factor K. It represents the number of consecutive frames processed by YOLO during the two semantic recognition operations based on CLIP. Based on the typical video frame rates of unmanned aerial vehicles (30 frames per second and 60 frames per second), we implemented a K + 1 scheduling scheme, such as the 29 + 1 and 59 + 1 schemes. The CLIP-based recognition function would be activated after every 29 or 59 YOLO detection frames. Under this strategy, the average per-frame processing latency $T_{avg}$ and the achievable scheduled frame rate $FPS_{sched}$ can be calculated as follows.

(13) $$\begin{array}{cc} T_{avg} & =T_{yolo}+\frac{1}{k}T_{clip} \end{array}$$

(14) $$\begin{array}{cc} FPS_{sched} & =\frac{1}{T_{avg}}=\frac{1}{T_{yolo}+\frac{1}{k}T_{clip}} \end{array}$$

With K increases, the computational overhead brought by the CLIP model is significantly distributed, and the overall performance of the system is infinitely close to the theoretical upper limit of pure YOLO detection.

It should be noted that in actual scenarios such as vast grasslands and areas with few wild animals, the changes in visual content are relatively slow and the target objects are scarce. Therefore, it is usually advisable to set the K value to be greater than 30. This not only reduces the system load but also significantly improves the response speed, while not affecting the integrity of semantic perception.

The frame-level adaptive scheduling mechanism is the core way to realize the efficient deployment of multi-modality models on the edge side, and its dynamic on-demand computing paradigm provides an important reference for similar computing-intensive applications on embedded platforms.

## 4. Experimental Simulation Analysis

### 4.1. Datasets Construction

#### 4.1.1. Construction

**Figure 5:**
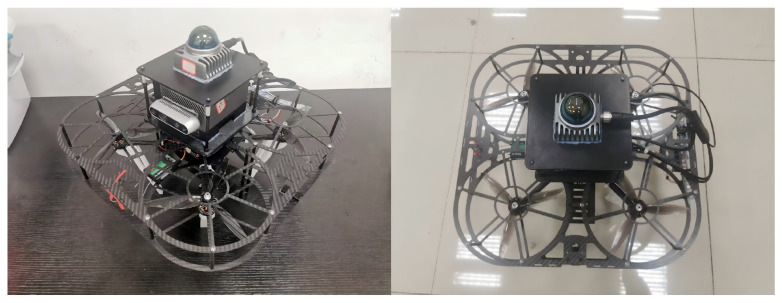
UAV-assisted data collection platform used for wildlife monitoring.

The datasets used in this study were collected using a self-built UAV-assisted acquisition platform. The platform consists of a quadcopter equipped with a global-shutter high-definition camera, an onboard Jetson Orin AGX processor, and a LiDAR sensor for autonomous navigation and spatial perception, as illustrated in Figure 5.

A total of 6700 images were collected in real-world field conditions. The training set consists of 5500 images, including 500 negative samples containing only background; the validation set contains 1200 images. Negative samples were included in the training set to enhance background recognition and reduce false positives. The dataset covers common African savanna wildlife species, including elephants, zebras, antelopes, wildebeests, giraffes, and buffaloes. Table 2 summarizes the category distribution of the constructed dataset.

All images were manually annotated with bounding boxes and corresponding category labels to ensure annotation accuracy and consistency. During data collection and selection, real application scenarios in UAV-assisted wildlife monitoring were fully considered, allowing the dataset to cover various typical field environments.

To fully demonstrate the recognition performance of the paper in complex environments such as with small targets and occlusions, the concepts of AP_sl_ and AP_ot_ are introduced in this article. Specifically, AP_sl_ represents the detection performance for small-scale targets. Objects whose bounding-box area is below 32 × 32 pixels. AP_ot_ represents the detection performance under the occlusion condition. In this study, an object is considered to be occluded when more than 30% of its visible area is covered by vegetation, terrain, shadows, or overlaps with other animals.

Overall, the dataset is suitable not only for assessing detection accuracy but also for analyzing environmental adaptability and generalization capability in complex scenarios. The dataset will be made publicly available upon acceptance.

In addition, to further verify the generalization capability of the proposed method, experiments are also conducted on the publicly available UAV-assisted wildlife dataset WAID [25], as described in Section 4.3. Unless otherwise specified, all experiments are conducted on the self-constructed UAV dataset.

#### 4.1.2. Evaluation Metrics

In this study, the mean average precision (mAP) is adopted as the primary evaluation metric, including mAP@0.5 and mAP@0.5:0.95, following standard object detection protocols. Precision (*P*) and recall (*R*) are defined as:

(15) $$\begin{array}{c} P=\frac{TP}{TP+FP} \end{array}$$

(16) $$\begin{array}{c} R=\frac{TP}{TP+FN} \end{array}$$

where TP, FP, and FN denote true positives, false positives, and false negatives, respectively.

For each category, the Average Precision (AP) is computed as the area under the Precision–Recall curve, and mAP is obtained by averaging AP over all categories.

(17) $$\begin{array}{c} AP=\int_{0}^{1}P(R)dR \end{array}$$

(18) $$\begin{array}{c} mAP=\frac{1}{N}\sum_{i=1}^{N}AP_{i} \end{array}$$

In addition to accuracy metrics, we further evaluate computational performance using Frames Per Second (FPS) to measure inference speed, as well as Floating Point Operations (FLOPs) and the number of parameters to assess model complexity.

#### 4.1.3. Hyperparameter Tuning for Foreground Proposal Generation

All experiments were conducted on a platform equipped with an Intel Core i9-12900KF CPU and an NVIDIA Tesla T10 GPU (16 GB VRAM). The models were implemented using PyTorch 2.5.0 with CUDA 12.4.

To determine optimal training configurations, we performed systematic hyperparameter tuning on key factors, including learning rate, input resolution, batch size, and loss weights,. Detailed hardware and software configurations are summarized in Table 3.

**Figure 6:**
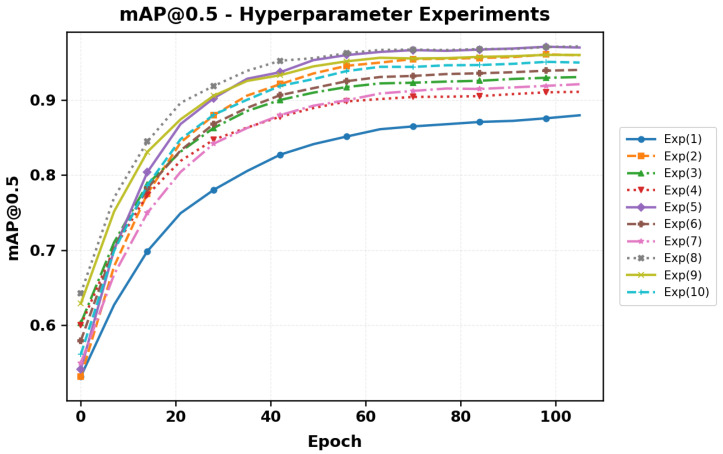
Graph of hyperparameter adjustments for the branch positioning only.

This section describes the process of adjusting the hyperparameters of the localization branch in YOLOv11. At this stage, the detector was optimized to extract candidate objects of foreground animals from the drone images, while the semantic recognition module based on CLIP was not involved. The experimental results are summarized in Table 3 and visually compared in Figure 6. As shown in Table 4 and Figure 6, the learning rate significantly affects convergence stability, where 0.001 achieves the best balance between accuracy and stable performance.

Increasing the input resolution refines detection performance, particularly for small objects, but introduces higher computational cost. A resolution of 960 × 960 provides a favorable trade-off between accuracy and efficiency. Batch size variations indicate that moderate settings (batch size = 8) yield more stable performance compared to smaller or larger values. Based on comprehensive evaluation, configuration No. 8 achieves the best overall performance, with mAP@0.5 of 0.97. Therefore, the final model adopts the following settings: learning rate =0.001, input size =960×960, batch size =8, with loss weights $\lambda_{box}=7.5$, $\lambda_{cls}=0.5$, and $\lambda_{dfl}=1.5$, while the IoU threshold is set to 0.5. It should be noted that the mAP@0.5 values reported in Table 3 only reflect the localization performance of the candidate boxes proposed by the detector, and do not represent the final end-to-end performance of the entire YOLIP framework.

### 4.2. Comparison Experiments

As shown in Table 5, the proposed YOLIP method outperforms all the other comparison methods in all the indicators, indicating that it has a significant advantage in the drone-assisted wildlife detection task.

Specifically, YOLIP attains a mAP@0.5 of 86.6%, outperforming YOLOv8m, YOLOv11n, and YOLO26n by 16.6%, 11.6% and 8.3%, respectively. Under the stricter mAP@0.5:0.95 metric, it also achieves consistent improvements. These results confirm the effectiveness of the proposed cross-modal alignment mechanism.

To further explain the source of the performance improvement, this paper conducts a gain decomposition analysis based on the results in Table 4. The comparison between YOLOv8n and YOLOv11n reflects the contribution brought about by the upgrade of the detector’s backbone. The complete YOLIP framework further increased the mAP@0.5 metric to 86.6%. The additional improvement mainly comes from the proposed YOLIP Head and IFM module, which respectively enhance the regional-level candidate representation and the cross-modal feature refinement.

**Figure 7:**
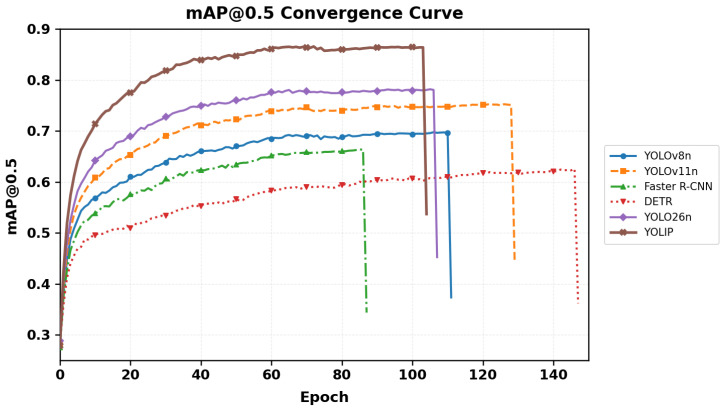
Visual model comparison chart.

**Figure 8:**
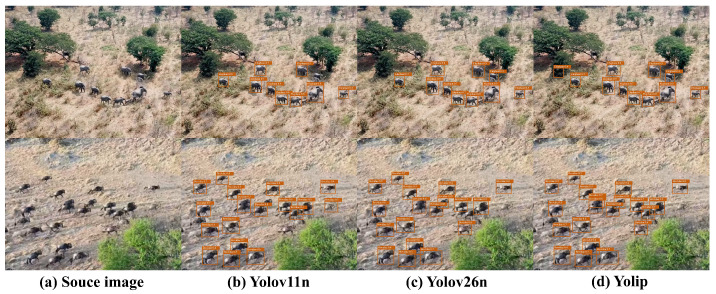
Migration scene comparison chart.

The convergence behaviors of different models are illustrated in Figure 7. It can be seen that the detection accuracy of YOLIP is significantly higher than that of other series of models, and it demonstrates stable and excellent training performance. Moreover, the qualitative comparison in complex transfer scenarios (Figure 8) further highlights its robustness and semantic understanding ability. In the transfer scenarios, the performance of YOLIP is highlighted.

**Figure 9:**
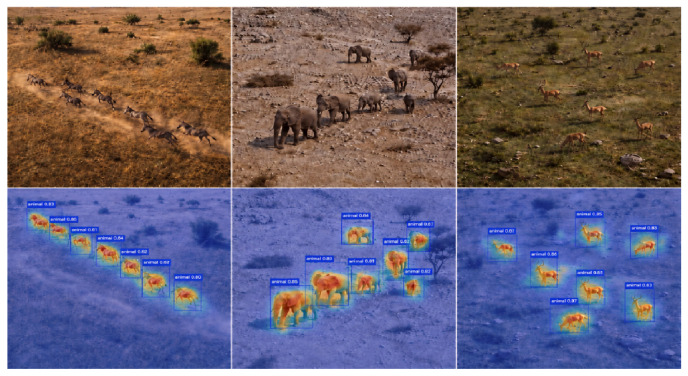
Grad-CAM visualization of YOLIP attention.

To further investigate the visual attention behavior of the proposed framework, Grad-CAM image visualization processing was applied to representative images of unmanned aerial vehicles observing wild animals, as shown in Figure 9.

The visualization results show that YOLIP has always focused on the biologically significant areas of the animals, rather than the irrelevant background areas, even in complex aerial scenes involving small targets, occlusions, and complex environmental textures. It must be pointed out that Grad-CAM reflects the spatial response of the YOLIP foreground localization stage to the target area, rather than the entire semantic alignment process itself.

### 4.3. Generalization on Public Datasets (WAID)

To further evaluate the generalization capability of the proposed YOLIP framework beyond the self-constructed UAV datasets, additional experiments are conducted on a publicly available aerial wildlife datasets, namely, WAID [24].

The WAID datasets consists of 14,375 aerial images captured by unmanned aerial vehicles (UAVs) in real-world wildlife monitoring scenarios. It contains six common large-animal categories, including cattle, sheep, and zebras, with full bounding box annotations provided for object detection tasks. Compared with our self-constructed datasets, WAID exhibits distinct characteristics in terms of object scale, viewpoint variation, and environmental diversity, making it a suitable benchmark for cross-dataset evaluation.

In this experiment, the proposed YOLIP model is directly applied to the WAID datasets with minimal fine-tuning to ensure fair comparison. No architectural modifications are introduced. Standard evaluation metrics, including mAP@0.5 and mAP@0.5:0.95, are adopted for performance assessment.

As shown in Table 6, YOLIP maintains competitive performance on WAID, demonstrating that the proposed feature alignment mechanism is not limited to a specific dataset but generalizes effectively to different UAV-assisted wildlife monitoring scenarios. YOLIP consistently outperforms baseline detectors, demonstrating robust cross-dataset generalization.

### 4.4. Ablation Study

#### 4.4.1. Ablation Study on IFM Dual-Pathway Design

To evaluate the contribution of the proposed dual-pathway IFM module, we conduct an ablation study by incrementally introducing its components. The results are summarized in Table 7.

**Figure 10:**
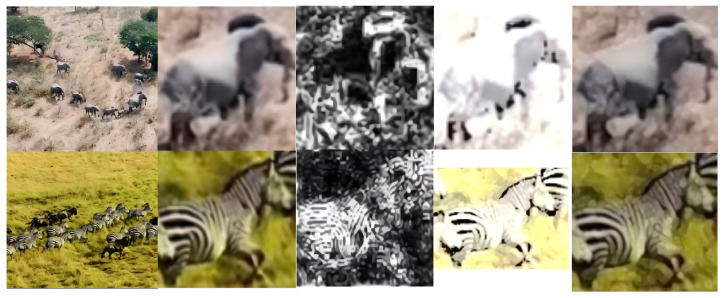
The geometric pathway effect figure.

As shown in Table 6, the naive YOLO-CLIP baseline, which directly resizes detected regions to 224×224 without any feature alignment, achieves 82.5% mAP@0.5. Introducing the geometric pathway alone—a learnable spatial transformer with reflection padding—improves mAP@0.5 to 83.2%, a gain of 0.7 percentage points. The enhancement is particularly notable on small objects, $AP_{sl}$ from 29.2% to 30.3%, confirming that learned geometric normalization preserves discriminative features that would otherwise be lost in naive resizing. The corresponding effect is illustrated in Figure 10 below.

Adding the semantic pathway (the weight-tied projection head $g_{\phi }^{\prime }$, without geometric alignment) yields 84.3% mAP@0.5, representing an improvement of 1.8 percentage points over the naive baseline. This demonstrates that contrastive semantic refinement alone provides meaningful gains, even when regions are not geometrically optimal.

The full IFM module, combining both geometric and semantic pathways, achieves 86.8% mAP@0.5, representing a 4.3 percentage point enhancement over the naive baseline. On the two challenging subsets, $AP_{s}l$ rises from 29.2% to 35.0% and $AP_{o}t$ from 34.3% to 42.1%. The synergistic gains—where the combined effect exceeds the sum of individual improvements—confirm that geometric normalization and semantic refinement are complementary: geometric alignment provides well-conditioned inputs for CLIP’s patch embedding, while semantic alignment ensures representational fidelity in the shared embedding space.

These results support the dual-pathway design of the IFM module. By jointly addressing the geometric and semantic domain gaps, the proposed alignment interface enables more effective cross-modal transfer between YOLO’s detection features and CLIP’s semantic space.

#### 4.4.2. Ablation Study on YH, FI, and FS Modules

To evaluate the contribution of each component in the proposed framework, we conduct a stepwise ablation study by incrementally introducing key modules into the baseline model. The results are summarized in Table 8 and Table 9. The baseline model (Model 1) adopts a conventional serial pipeline, where YOLOv11 is used for detection and CLIP is applied independently for recognition, achieving a mAP@0.5 of 82.5%. Model 2 introduces the YOLIP Head (YH), which generates language-aligned proposal embeddings embedding and enhances feature representation at the detection stage. This results in a 3.4% improvement in mAP@0.5, demonstrating the effectiveness of early-stage semantic alignment. Model 3 further incorporates the IFM module, enabling cross-modal alignment between visual features and CLIP embeddings.

This increases mAP@0.5:0.95 from 57.6% to 60.7%, indicating enhanced semantic consistency and generalization capability. Finally, Model 4 integrates a frame-adaptive scheduling (FS) mechanism that decouples detection and semantic recognition through asynchronous inference scheduling. While maintaining comparable detection accuracy, the proposed scheduling strategy significantly increases the stream-level processing throughput from 11.2 FPS in a fully serial pipeline to 57.8 FPS under asynchronous deployment. These results demonstrate that each module contributes positively to the overall performance. In particular, the YOLIP Head improves detection quality, the FI module enhances semantic alignment, and the FS strategy significantly boosts computational efficiency.

### 4.5. Comprehensive Comparison with YOLO-World

To further evaluate the effectiveness of the proposed framework, we conduct a comprehensive comparison with YOLO-World, a representative open-vocabulary detection paradigm based on YOLO and CLIP. The comparison focuses on architecture design, training paradigm, efficiency, and real-world applicability.

As summarized in Table 10, the two methods differ fundamentally in design philosophy. YOLO-World follows a “semantics-first” paradigm by aligning detector features with text embeddings, while YOLIP adopts a “detection-first, semantics-refined” strategy, prioritizing high-recall proposal generation followed by semantic refinement. The quantitative results in Table 11 demonstrate that YOLIP achieves superior closed-set detection performance, reaching 86.6% mAP@0.5, which surpasses YOLO-World by 5.48%. This confirms the effectiveness of the forward pipeline design in practical detection tasks.

Although YOLO-World exhibits stronger zero-shot generalization due to its end-to-end alignment training, YOLIP provides a more stable and efficient solution for real-world deployment. Furthermore, the asynchronous scheduling strategy enables YOLIP to decouple detection and recognition processes, reducing redundant computation in video streams. This design makes it particularly suitable for resource-constrained edge environments and real-time applications.

In general, while YOLO-World emphasizes semantic generalization, YOLIP offers a better balance between accuracy, efficiency, and deployability, making it more practical for UAV-assisted wildlife monitoring scenarios.

## 5. Conclusions

This paper presents YOLIP, a hierarchical perception framework that effectively integrates YOLO-based detection with CLIP-based semantic recognition through a unified cross-modal alignment pipeline. By introducing the YOLIP Head and the Interaction Fusion Module (IFM), the proposed method enables the generation of language-aligned proposals and fine-grained semantic refinements, achieving robust performance in both detection accuracy and open-vocabulary recognition.

In addition, the proposed frame-level scheduling and asynchronous inference strategy significantly improves computational efficiency in continuous video streams, making the framework well suited for real-time deployment in UAV-assisted wildlife monitoring scenarios.

Although this framework has many of the aforementioned advantages, it still has some limitations. Firstly, the semantic recognition ability of YOLIP still relies on the quality of the proposals generated by the detector. This means that the foreground regions missed during the localization stage may propagate errors to the subsequent semantic refinement stage. Secondly, although CLIP enhances the semantic representation capability, its computational cost is still relatively high compared to lightweight detection models. This may limit the deployment efficiency on low-power embedded hardware. Furthermore, the current asynchronous scheduling strategy relies on manually predefined scheduling intervals and trigger thresholds, which may not achieve the optimal generalization effect under different video dynamics and environmental conditions.

Furthermore, although the proposed framework employs semantic alignment technology based on CLIP, this study primarily focuses on closed wildlife monitoring scenarios rather than a completely open vocabulary detection benchmark. The generalization ability of this framework when dealing with novel and intricate object categories still requires further investigation.

## Figures and Tables

**Table 1:** Comparison of execution modes in the YOLIP framework.

Mode	Parallelism	Latency Characteristic	Throughput	Suitable Scenarios
Serial Mode	Fully sequential	Accumulated across all stages	Limited	Real-time, low-latency systems
Asynchronization Mode	Pipeline paralle	Dominated by slowest stage	High	Video streaming, offline analysis

**Table 2:** Category distribution of the self-built UAV wildlife dataset.

Category	Training Images	Validation Images	Instances
Elephant	1080	230	3125
Zebra	980	210	4287
Antelope	920	195	3654
Wildebeest	760	170	2813
Buffalo	640	145	2146
Giraffe	620	140	1879
Background-only	500	110	–
Total	5500	1200	17,904

**Table 3:** Experimental environment and training configuration.

Category	Item	Specification
Hardware	CPU	Intel Core i9-12900KF
	GPU	NVIDIA Tesla T10 (16 GB VRAM)
	RAM	32 GB DDR5
Software	Operating System	Ubuntu 22.04 LTS
	Deep Learning Framework	PyTorch 2.5.0

**Table 4:** Detector-only hyperparameter tuning for the YOLOv11 localization branch.

Exp.	LR	Input Size	Batch	IoU	Box	Cls	DFL	mAP@0.5	FPS
1	0.0003	960×960	8	0.50	7.5	0.5	1.5	0.88	64
2	0.001	960×960	8	0.50	15	0.5	1.5	0.96	68
3	0.003	960×960	8	0.50	7.5	0.5	1.5	0.93	61
4	0.001	640×640	8	0.50	7.5	0.5	1.5	0.91	84
5	0.001	1280×1280	8	0.50	7.5	0.5	1.5	0.97	39
6	0.001	960×960	4	0.50	7.5	0.5	1.5	0.94	65
7	0.001	960×960	16	0.50	7.5	0.5	1.5	0.92	66
8	0.001	960×960	8	0.50	7.5	0.5	1.5	0.97	67
9	0.001	960×960	8	0.50	7.5	1.0	1.5	0.96	67
10	0.001	960×960	8	0.55	15	0.5	1.5	0.95	67

**Table 5:** Comparative analysis of the performance of the YOLIP model and mainstream detectors.

Model	mAP@0.5	mAP@0.5:0.95	FPS *	Params (M)	AP_sl_
YOLOv8n	70.0	47.0	18.2	7.6	0.31
YOLOv11n	75.0	49.4	30.3	8.8	0.34
Faster R-CNN	67.0	42.0	6.5	41.5	0.23
DETR	63.0	41.0	5	41.0	0.20
YOLO26n	78.3	50.1	42.0	8.4	0.34
YOLIP	86.6	60.7	57.8	153.8	0.35

* The FPS of YOLIP is measured under asynchronous scheduling mode rather than pure serial inference.

**Table 6:** Generalization performance of YOLIP on the WAID datasets.

Model	mAP@0.5	mAP@0.5:0.95
YOLOv11n	61.4	42.8
YOLO26n	64.2	45.6
YOLIP	72.1	56.7

**Table 7:** Ablation study on the geometric and semantic pathways of IFM.

Model	mAP@0.5 (%)	AP_sl_	AP_ot_
YOLO-CLIP	82.5	29.2	34.3
+Geometric Pathway only	83.2	30.3	36.8
+Semantic Pathway only	84.3	32.2	39.7
+IFM (Geometric + Semantic)	86.8	35.0	42.1

**Table 8:** Ablation study on YH and IFM.

Model	YH	IFM	FLOPs/G	P/%	R/%	mAP@0.5/%	mAP@0.5:0.95/%	FPS
1			156.8	80.3	78.1	82.5	57.6	11.2
2	√ ^*^		160.3	83.4	80.7	85.9	59.5	19.5
3	√	√	163.2	84.1	81.5	86.8	60.1	18.8

* √ indicates that the module is used.

**Table 9:** Throughput analysis under frame-adaptive scheduling.

Scheduling Mode	Effective Throughput FPS
Full serial pipeline	18.8
Asynchronous scheduling (k = 30)	57.8

**Table 10:** A multi-dimensional comparison between YOLIP and YOLO-World.

Dimensions	YOLO-World	YOLIP
Visual Branch	Open-vocabulary detection via region-to-text alignment in a shared vision-language embedding space	Forward semantic recognition using CLIP visual encoder for fine-grained classification of candidate regions
Semantic Branch	Open-vocabulary via reverse matching	Fine-grained via forward recognition.
Training Paradigm	End-to-end joint optimization with cross-modal alignment loss for zero-shot generalization	Two-stage training: supervised detection training followed by frozen CLIP-based semantic refinement
Design Philosophy	Semantics-driven detection: adapts detector features to align with semantic concepts	Detection-driven semantic refinement: prioritizes high-recall proposal generation and refines them via semantic alignment

**Table 11:** YOLIP vs. YOLO-World comparison.

Model	mAP@0.5	mAP@0.5:0.95	AP_sl_	AP_ot_	FPS (Serial) *	FPS (Async) *	FLOPs (G)
YOLO-World	82.1	48.9	0.47	0.64	41.5	–	160.0
YOLIP (ours)	86.6	60.7	0.45	0.64	18.8	57.8	163.4

* FPS (serial): serial mode. FPS (async): asynchronous mode (k = 30).

## Data Availability

The publicly available dataset is obtained from the website https://doi.org/10.3390/app131810397. Other data used to support the findings of this study are available from the corresponding author upon reasonable request.

## Abbreviations

The following abbreviations are used in this manuscript:

YOLO	You Only Look Once
YH	YOLIP Head
CLIP	Contrastive Language-Image Pre-training
UAV	Unmanned Aerial Vehicle
RoIAlign	Region of Interest Align
IFM	Interaction Fusion Module
mAP	mean Average Precision
AP	Average Precision
FPS	Frames Per Second
FLOPs	Floating Point Operations
TP	True Positives
FP	False Positives
